# The increase of methicillin-resistant *Staphylococcus aureus *(MRSA) and the presence of an unusual sequence type ST49 in slaughter pigs in Switzerland

**DOI:** 10.1186/1746-6148-7-30

**Published:** 2011-06-24

**Authors:** Gudrun Overesch, Sabina Büttner, Alexandra Rossano, Vincent Perreten

**Affiliations:** 1 Institute of Veterinary Bacteriology, Vetsuisse Faculty, University of Bern, Länggass-Strasse 122, CH-3012 Bern, Switzerland; 2 Centre for Zoonoses, Animal Bacterial Diseases and Antibiotic Resistance (ZOBA), Institute of Veterinary Bacteriology, Vetsuisse Faculty, University of Bern, Länggass-Strasse 122, CH-3012 Bern, Switzerland; 3 Federal Veterinary Office, Schwarzenburgstrasse 155, CH-3003 Bern, Switzerland

## Abstract

**Background:**

In years past, methicillin-resistant *S. aureus *(MRSA) has been frequently detected in pigs in Europe, North America and Asia. Recent, yet sporadic studies have revealed a low occurrence of MRSA in Switzerland. In 2009, a monitoring survey of the prevalence and genetic diversity of methicillin-resistant *S. aureus *(MRSA) in slaughter pigs in Switzerland was conducted using methods recommended by the EU guidelines, and using a sampling strategy evenly distributed throughout the year and representative of the Swiss slaughter pig population. Monitoring should determine if the overall prevalence of MRSA in the entire country is increasing over the years and if specific multi-resistant MRSA clones are spreading over the country.

**Results:**

In 2009, the nasal cavities of eight out of 405 randomly selected pigs were positive for MRSA, representing a prevalence of 2.0% (95% CI 0.9-3.9). The following year, 23 out of 392 pigs were positive for MRSA [5.9% prevalence (95% CI 3.8-8.7)]. Three multilocus sequence types (ST), four *spa *types and two types of staphylococcal cassette chromosome *mec *(SCC*mec*) elements were detected. The most frequent genotypes were ST398 (MLST)-(*spa*)t034-V(SCC*mec*) (n = 18) and ST49-t208-V (n = 7), followed by ST398-t011-V (n = 4), ST398-t1451-V (n = 1), and ST1-t2279-IVc (n = 1). The isolates displayed resistance to ß-lactams [*mecA*, (31/31); *blaZ*, (19/31)]; tetracycline [*tet*(M), (31/31); *tet*(K), (30/31)] (n = 31); macrolides and lincosamides [*erm*(C) (4/31) or *erm*(A) (18/31)] (n = 22); tiamulin [*vga*(A)*v *(9/31) or unknown mechanism (18/31)] (n = 27); trimethoprim [*dfr*(G) (18/31); spectinomycin [*ant(9)-Ia *(19/31) or unknown mechanism (3/31)] (n = 22); streptomycin [*str *(19/31)]; sulphamethoxazole (7/31) and ciprofloxacin (n = 1) (mechanisms not determined).

**Conclusions:**

This study is the first to describe the presence of MRSA ST49 in slaughter pigs, and to demonstrate a significant and nearly three-fold increase of MRSA prevalence in pigs within two years. The presence of a specific clonal lineage of MRSA from Switzerland suggests that it has been selected in Swiss pig husbandry. Effective hygiene measures should be enhanced within the entire pig production chain to suppress the spread of these pathogens into the community.

## Background

Over the years, methicillin-resistant *Staphylococcus aureus *(MRSA) has been increasingly reported in animals worldwide [1-4]. A specific clonal lineage, MLST ST398, has been found to be widespread among pigs in Europe and North America, whereas ST9 seemed to be predominant in Asia [4-6]. Several *spa *types were detected within these lineages with *spa *types t011, t108, and t034 being the most frequent in MRSA ST398, and t899 in MRSA ST9 [3-6]. Colonisation with MRSA ST398 was also described in other animals like poultry, horses, and calves, and recently, ST398 has been associated with infections in animals and humans [2,4,7]. Humans that come in close contact with farm animals were more likely to be colonised with livestock-associated ST398 and had a higher risk of developing infections with ST398 in case of hospitalisation [8-18]. The pig husbandry environment also represents a large reservoir for MRSA. MRSA ST398 was recovered from dust samples in pig production holdings in Europe with an average prevalence of 26.9% varying between 0% and 51.2% among the EU member states in 2008 [19]. MRSA ST398-t011 was the most dominant type (63.5%), followed by ST398-t108 (9.4%), and ST398-t034 (8.4%). 1.4% of the MRSA from this study were from seven other non-ST398 strains (ST1, ST5, ST8, ST9, ST39, ST97, and ST132). The majority of the non-ST398 strains originated from Germany and Italy [19].

In 2008, dust samples from pig husbandries in Switzerland were analysed and did not contain MRSA [19]. In fact, there was no MRSA detected during previous studies in Swiss slaughter pigs and pig carcasses before 2009 [20,21]. However in 2009, 10 MRSA ST398-t034, which contained the staphylococcal cassette chromosome SCC*mec*V (ST398-t034-V), were found among 800 nasal swabs from Swiss pigs at two slaughterhouses; these results indicated that MRSA had also emerged in the Swiss pig population [22]. That same year, an official monitoring of MRSA in pigs at the slaughterhouse was launched to determine the overall prevalence in the entire country. A sampling strategy for monitoring antimicrobial resistance was developed in 2008, which considered the geographical distribution and size of the slaughterhouses, and the number of slaughtered pigs in Switzerland [23]. Since 2009, this sampling plan was also used for monitoring MRSA. This study will determine the overall prevalence and dynamics of MRSA colonisation in the slaughtered pig population and will indicate if specific genetic lineages of MRSA are colonising pigs in Switzerland.

## Results

### Increased prevalence and molecular typing

**Figure 1:**
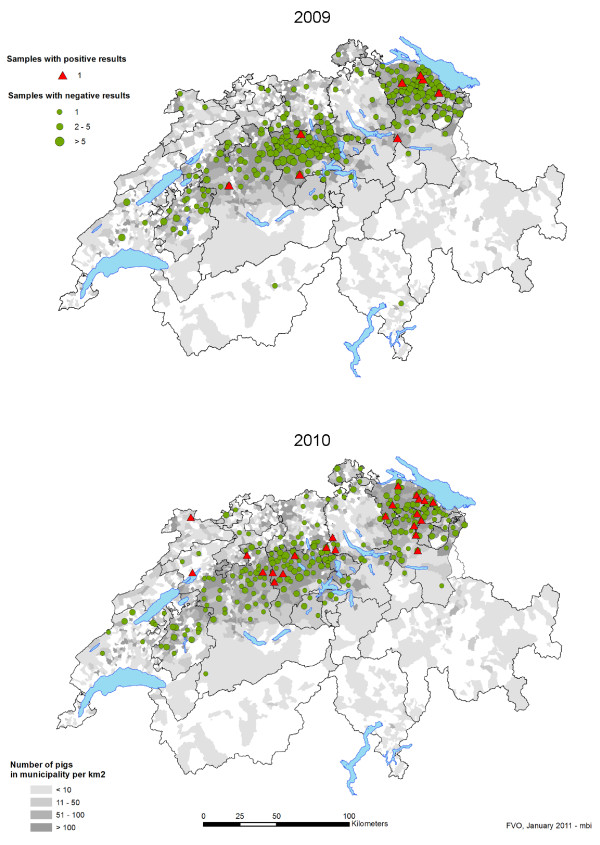
**Pig density and geographical distribution of the samples and results for MRSA isolated from the nasal cavities of slaughter pigs in Switzerland in 2009 and 2010**. The pig density appears in grey. Samples with positive results appear as red triangles, samples with negative results appear as green dots.

MRSA (n = 31) were detected in samples from 5 of 9 slaughterhouses (A, B, C, E, G) in 2009 and in 7 of 9 slaughterhouses (A-D, G-I) in 2010 (Table 1). MRSA was mostly found in pigs raised in cantons where the pig population is the highest (Figure 1), that is, Thurgau (n = 11), Lucerne (n = 6), St. Gallen (n = 5), Bern (n = 4), and Aargau (n = 2). Single isolates were found in pigs from Appenzell Innen Rhodes, Jura, and Zurich (Table 1 and Table 2). In 2009, the prevalence of MRSA in Swiss slaughter pigs was 2.0% (95% CI 0.9-3.9) with eight out of 405 pig nasal samples being positive and increased significantly (p-value < 0.05) to 5.9% (95% CI 3.8-8.7) in 2010 with 23 out of 392 nasal swabs containing MRSA. MRSA was detected every month in 2010, except in September, with up to five positive samples per month, whereas MRSA was only sporadically detected over the year in 2009.

**Table 1:** Distribution of methicillin-resistant *Staphylococcus aureus *genotypes in slaughterhouses and pig farms in Switzerland.

	**2009**					**2010**				
		
**Abattoir**	**Total no. of** **samples**	**No. of** **MRSA** **positive** **samples**	**Percentage** **of MRSA** **positive** **samples**	**Origin^a^**	**Genotype (n)**	**Total no. of** **samples**	**No. of** **MRSA** **positive** **samples**	**Percentage** **of MRSA** **positive** **samples**	**Origin^a^**	**Genotype (n)**
		
A	84	2	2.4	TG1, LU2	ST398-t011-V (2)	68	11	16.2	LU4, LU5, SG3, SG5, TG5, TG8, TG9, BE3, BE4 ZH1, JU1	ST398-t034-V (11)
B	90	1	1.1	SG1	ST398-t011-V	101	2	2.0	AG2, SG6	ST49-t208-V (2)
C	63	1	1.6	BE1	ST398-t034-V	65	4	6.2	TG6 BE2, TG7, LU6	ST49-t208-V ST398-t034-V (3)
D	62	0	0	-	-	58	2	3.4	AG1, TG10	ST398-t034-V (2)
E	39	1	2.6	LU1	ST398-t1451-V	37	0	0	-	-
F	28	0	0	-	-	22	0	0	-	-
G	19	3	15.8	TG3, TG2, SG2	ST1-t2279-IVc ST49-t208-V (2)	15	2	13.3	TG4, SG4	ST49-t208-V (2)
H	10	0	0	-	-	14	1	7.1	LU3	ST398-t034-V
I	10	0	0	-	-	12	1	8.3	AI1	ST398-t011-V
					
A-I (all)	405	8	2.0			392	23	5.9		

a) Geographical origin of the farms: letters indicate the Cantons (AI, Appenzell Innen Rhodes; AG, Aargau; BE, Bern; JU, Jura; LU, Luzern; SG, St Gallen; TG, Thurgau; ZH, Zurich). and numbers indicate individual farms.

**Table 2:** The clonal lineage and resistance profile of methicillin-resistant *Staphylococcus aureus *from pig noses in Switzerland.

						Antibiotic resistance genes and resistance breakpoints (mg/L)^b^										
						
MLST	*spa *type	SCC*mec *type	Number of isolates	Farm^a^	Representative strain	FOX	PEN	TET	ERY	CLI	STR	TIA	SPC	TMP	SMX	CIP
						
						(>4)	(>0.125)	(>2)	(>2)	(>0.5)	(>16)	(>2)	(>128)	(>2)	(>128)	(>1)
**2009**																
ST1	t2279	IVc	1	TG3	IMD887-09	*mecA*	*mecA blaZ*	*tet*(M)	*erm*(A)	*erm*(A)			*ant(9)-Ia*			+
ST398	t011	V	1	SG1	IMD358-09	*mecA*	*mecA*	*tet*(M) *tet*(K)				*vga*(A)*v*				
ST398	t011	V	1	TG1	IMD355-09	*mecA*	*mecA*	*tet*(M) *tet*(K)	*erm*(C)	*erm*(C)			+			
ST398	t011	V	1	LU2	IMD1753-09	*mecA*	*mecA*	*tet*(M) *tet*(K)					+			
ST398	t034	V	1	BE1	IMD1116-09	*mecA*	*mecA blaZ*	*tet*(M) *tet*(K)	*erm*(A)	*erm*(A)	*str*	+	*ant(9)-Ia*	*dfr*(G)		
ST398	t1451	V	1	LU1	IMD1270-09	*mecA*	*mecA*	*tet*(M) *tet*(K)	*erm*(C)	*erm*(C)	*str*	*vga*(A)*v*				
ST49	t208	V	1	TG2	IMD1000-09	*mecA*	*mecA*	*tet*(M) *tet*(K)	*erm*(C)	*erm*(C)		*vga*(A)*v*			+	
ST49	t208	V	1	SG2	IMD1771-09	*mecA*	*mecA*	*tet*(M) *tet*(K)				*vga*(A)*v*			+	

**2010**																
ST398	t034	V	16	TG5 TG8 TG9 TG10 LU3 LU4 LU5 LU6 BE2 BE3 BE4 AG1 JU1 SG3 SG5 ZH1	IMD49-10	*mecA*	*mecA blaZ*	*tet*(M) *tet*(K)	*erm*(A)	*erm*(A)	*str*	+	*ant(9)-Ia*	*dfr*(G)		
ST398	t034	V	1	TG 7	IMD704-10	*mecA*	*mecA blaZ*	*tet*(M) *tet*(K)	*erm*(A)	*erm*(A)		+	*ant(9)-Ia*	*dfr*(G)		
ST398	t011	V	1	AI1	IMD233-10	*mecA*	*mecA*	*tet*(M) *tet*(K)	*erm*(C)	*erm*(C)			+			
ST49	t208	V	3	SG4 SG6 TG4	IMD426-10	*mecA*	*mecA*	*tet*(M) *tet*(K)				*vga*(A)*v*			+	
ST49	t208	V	1	TG6	IMD603-10	*mecA*	*mecA*	*tet*(M) *tet*(K)	*erm*(A)	*erm*(A)		*vga*(A)*v*			+	
ST49	t208	V	1	AG2	IMD2002-10	*mecA*	*mecA*	*tet*(M) *tet*(K)			*str*	*vga*(A)*v*			+	

a) Geographical origin of the farm: letters indicate the Cantons (AI, Appenzell Innen Rhodes; AG, Aargau; BE, Bern; JU, Jura; LU, Luzern; SG, St Gallen; TG, Thurgau; ZH, Zurich). and numbers indicate individual farms.

b) CIP, ciprofloxacin; CLI, clindamycin; ERY, erythromycin; FOX, cefoxitin; PEN, penicillin; SPC, spectinomycin; SMX, sulphamethoxazole; STR, streptomycin; TET, tetracycline; TIA, tiamulin; TMP, trimethoprim. The MIC breakpoints (in micrograms per millilitre) that determine resistance were recommended from EUCAST for *S. aureus *http://www.eucast.org. Resistance breakpoints for tiamulin, spectinomycin, streptomycin and sulphamethoxazole were tentatively derived from epidemiological MIC cut-off values from EUCAST.

Antibiotic resistance genes and their functions are indicated as follows: *ant(9)-Ia*, spectinomycin adenylnucleotidyltransferase; *bla*Z, β-lactamase; *dfr*(G), dihydrofolate reductase; *erm*(A) and *erm*(C), macrolides, lincosamides and streptogramins B rRNA methylase; *mecA*, penicillin-binding protein PBP2a; *str*, streptomycin adenyltransferase; *tet*(K), tetracycline efflux protein; *tet*(M), tetracycline ribosomal protection protein; *vga*(A)*v*, pleuromutilins and streptogramins A ATP binding transporter.

ND, not detected; +, The MIC values were greater than the resistance breakpoint, but the resistance mechanism remained uncharacterised; blank spaces indicate no resistance

With the exception of one isolate (SCC*mec*IVc), most of the MRSA contained the SCC*mec*V element (Table 2). The eight MRSA isolated in 2009 belonged to five genotypes: ST398-t011-V (n = 3), ST398-t034-V (n = 1), ST398-t1451-V (n = 1), ST49-t208-V (n = 2), and ST1-t2279-IVc (n = 1); each isolate displayed a different resistance profile (Table 2). In 2010, only three clonal lineages were identified with ST398-t034-V (n = 17) and ST49-t208-V (n = 5) being the most common, whereas ST398-t011-V was only detected once (Table 1 and Table 2).

MRSA isolated from a same slaughterhouse in 2009 and 2010 displayed a different genetic profile and were isolated at different days, except for some MRSA ST398-t034 and ST49-t208 isolates. They were also detected several times from samples from the same slaughterhouses at different days [slaughterhouses A, C, D (ST398-t034)] as well as during the same sampling day in 2010 [slaughterhouses A (ST398-t034) and G (ST49-t208)]. All samples originated from pigs raised in different husbandry (Table 1 and Table 2).

### Resistance profiles

Isolates belonging to the most commonly detected genotype, ST398-t034-V, shared the same resistance profile; however, one isolate was susceptible to streptomycin and did not contain the streptomycin adenyltransferase gene *str *(Table 2). Otherwise, ST398-t034-V isolates showed resistance to ß-lactams specified by *mecA *and *blaZ*, tetracycline [*tet*(K), *tet*(M)], macrolide-lincosamide-streptogramin B (MLS_B_) antibiotics [*erm*(A)], spectinomycin [*ant(9)-Ia*], streptomycin [*str*], trimethoprim [*dfr*(G)], and tiamulin. The tiamulin resistance could not be attributed to either of the five known pleuromutilin-streptogramin A resistance genes *vga*(A), *vga*(A)*v, vga*(B), *vga*(C), *vga*(D) [24], or to the *cfr *rRNAmethylase transferase gene which confers cross resistance to phenicols, lincosamides, oxazolidones, pleuromutilins, and streptgramin A antibiotics [25]. Mutations in the 23 rRNA and *rplC *gene were not investigated [26].

Different resistance profiles were observed among the ST49-t208-V isolates, which was the second most prevalent lineage. The difference was mainly due to the presence, or absence, of either of the MLS_B _resistance genes *erm*(A) or *erm*(C) (Table 2). In general, ST49 isolates displayed resistance to ß-lactams [*mecA*], tetracycline [*tet*(K), *tet*(M)], tiamulin [*vga*(A)*v*], and sulphonamides (these mechanisms were not characterised). Notably, ST49-t208 isolates were susceptible to trimethoprim and resistant to sulphonamides, whereas ST398-t034 isolates were resistant to trimethoprim and susceptible to sulphonamides.

Different antibiotic resistance profiles were also found among the less frequent isolates, ST398-t011 and ST398-t1451. They all displayed resistance to ß-lactams [*mecA*] and tetracycline [*tet*(K), *tet*(M)]; however, resistance to MLS_B _antibiotics [*erm*(C)], tiamulin [*vga*(A)*v*], streptomycin [*str*], and spectinomycin varied from strain to strain (Table 2). The ST1-t2279-IVc isolate was only detected once and showed resistance to ß-lactams [*mecA, blaZ*], tetracycline [*tet*(M)], MLS_B _antibiotics [*erm*(A)], and spectinomycin [*ant(9)-Ia*]; additionally, a resistance to ciprofloxacin was detected (Table 2). The resistance mechanism to fluoroquinolones which may be due to mutations in the topoisomerase genes [27] was not determined. In contrast, MRSA isolates from 2009 and 2010 were susceptible to vancomycin, gentamicin, kanamycin, chloramphenicol, quinupristin/dalfopristin, fusidic acid, mupirocin, rifampicin, and linezolid; importantly, none of the isolates carried the Panton-Valentine Leukocidin toxin.

## Discussion

### Molecular typing and epidemiology

This study was the first to reveal the presence of MRSA ST49 in pigs. MRSA ST49 was already present in 2009, but its prevalence increased in 2010. The presence of the new clonal lineage ST49-t208, which is unique to MRSA from pigs in Switzerland, suggests that the selection occurred within the Swiss pig population. This idea is supported by the fact that MRSA ST49 was detected in seven different pigs from seven different farms slaughtered in three different slaughterhouses, and that ST49 isolates displayed different resistance profiles mainly due to the acquisition of an additional *erm*(A) or *erm*(C) gene (Table 2). The *spa *type t208, which is associated with ST49, was previously found in seven out of 48 methicillin-susceptible *S. aureus *MSSA from pigs in Switzerland [20], suggesting a possible acquisition of the SCC*mec *element. ST49 is rarely mentioned in the literature; it was not detected among 2890 MSSA and MRSA from human infections in the 26 EU countries, the 133 *S. aureus *colonising healthy adults in Switzerland nor within the 572 MSSA isolates from Swiss children [28-30]. ST49 has only been associated with MSSA involved in one human infection in the United Kingdom and in three cases of skin lesions and laryngeal ulceration in wild squirrels [31,32]. In the latter three cases, the authors mentioned close contact with persons who fed the squirrels as a possible source of infection. Further screening of humans, including people working with food-producing animals, would be necessary to determine if humans also play a role in the dissemination of MRSA ST49, as demonstrated with ST398. Human carriage of ST398 has been widely documented in other European countries, and the exchange of ST398 between humans and pigs has been reported in the Netherlands and Denmark [13,33,34]. Furthermore, ST398-t034 was the most predominant lineage found in Swiss pigs, and has been detected in one out of 133 veterinarians in Switzerland [22]. In our study, ST1 was only detected once, although it has been associated with MRSA infections in humans in the United Kingdom and Spain, and in horses in Austria [31,35,36]. ST1 represented, however, 15.6% of the MRSA-positive pig finishing holdings in Italy [37]. Cases of transmission of ST1 between humans and cows have also been reported [38].

Transmission of MRSA among pigs during animal transport and at slaughterhouse has been described [39]. In our study, no direct association of STs to a specific abattoir has been observed, except for slaughterhouse A, where ST398-t034 has been predominantly detected in the samples. Transmission which may have occurred at slaughterhouse may not be excluded for this abattoir. Otherwise, MRSA ST398 and ST49 were found in different slaughterhouses at different sampling dates and the pigs originated from different farms.

### Increase of MRSA prevalence

This study is also the first to describe MRSA prevalence in slaughter pigs in Switzerland using a sampling plan which was representative of the Swiss slaughter pig population and which was evenly distributed over time. This strategy allowed us to obtain statistically significant and representative data for the evaluation of the chronological distribution of MRSA. Such representative sampling criteria were not considered in the two previous studies reporting MRSA in pigs in Switzerland, resulting in a less accurate estimation of the prevalence over the entire country [20,22]. However, these studies indicated that the prevalence of MRSA was low among the proportion of pigs analysed before 2009. Our study also showed that the prevalence of MRSA in 2009 was low, and indicated an approximate three-fold increase within one year. The increased diversity of MRSA and the emergence of the clonal lineage ST49 unique to MRSA from Swiss pigs suggested that MRSA-positive pigs imported into Switzerland have not introduced this MRSA. However, Switzerland sporadically imports a low number of breeding pigs, from which MRSA ST398 may have been introduced. In 2010, for example, Switzerland imported pigs from Denmark (n = 10), The Netherlands (n = 4), and Germany (n = 1), where the prevalence of MRSA ST398 is high (Federal Veterinary Office, pers. communication). Like Switzerland, the predominant clone in Denmark and Germany is ST398-t034, whereas MRSA ST398-t011 is the predominant in The Netherlands [40-42].

## Conclusions

Two predominant clonal lineages, ST398-t034 and ST49-t208, are spreading within the Swiss slaughter pig population. Focus should be stressed on prevention of the transmission of these multi-resistant zoonotic bacteria from the farms into the community. Human carriage of MRSA represents a new challenge for public health because people that are in the vicinity of animals have been shown to have a higher risk of developing a MRSA infection when hospitalised. Targeted screening for MRSA in at-risk people, that is, farmers, veterinarians, and other people with close contact to animals, as recommended by Harbarth et al. (2011) [43], should be taken into account upon hospital admission. A study on MRSA decolonisation in humans with close contact to pigs showed a low efficacy due to continuous re-colonisation [44]. Therefore, effective hygiene measures into the entire pig production chain should be maintained as proposed by the Scientific Advisory Group on Antimicrobials [8]. Nevertheless, periodic monitoring of the MRSA in livestock animals at slaughterhouses is now established in Switzerland at the National Reference Laboratory for Antimicrobial Resistance (Centre of Zoonoses, Bacterial Animal Diseases and Antimicrobial Resistance (ZOBA), Institute of Veterinary Bacteriology, Vetsuisse Faculty, University of Bern), for the surveillance of MRSA in the livestock population.

## Methods

### Sampling

Representative samples were taken in accordance with the framework of a national monitoring program on antimicrobial resistance in food animals [23]. A minimum required sample size of 382 randomly selected animals was calculated with the assumptions of an infinite population size, a prevalence of 5%, a desired confidence level of 97.5% and an accuracy of 2.5%. The samples were randomly taken at the nine biggest abattoirs (A to I), where over 85% of the pigs in Switzerland were slaughtered. Only one sample was taken per animal holding. The number of samples in the sampling frame collected from each slaughterhouse was proportional to the number of pigs slaughtered at each establishment per year. One to 8 samples were taken per sampling day and abattoir. The samples collected on Mondays and Tuesdays were equally distributed over the years. Based on these data, random sampling plans were conducted, resulting in a total of 405 nasal swabs of fattening pigs in 2009 and 392 swabs from pigs in 2010.

### Isolation of MRSA

Samples were taken using transport swabs (Oxoid Ltd, Basingstoke, England) from the nares of the pigs subsequent to stunning by officials of the Swiss abattoir authorities and immediately after sampling, were transported to the laboratory without cooling. After arrival, swabs were transferred into tubes containing 10 ml Mueller Hinton Broth supplemented with 6.5% NaCl and incubated aerobically at 37°C for 24 h under agitation. One ml from this pre-enrichment was inoculated into 9 ml tryptone soy broth containing 3.5 mg/L cefoxitin and 75 mg/L aztreonam, and further incubated aerobically at 37°C for 24 h. A loopful was then spread onto MRSA selective agar plates (BBL ™ CHROMagar ™ MRSA; Becton Dickinson, Franklin Lakes, NJ), which were incubated at 37°C for 24 h. Pink to mauve-colored colonies were regarded as suspicious and five presumptive colonies were cultivated onto tryptone soy agar plates containing 5% sheep blood (TSA-SB) (Oxoid Ltd, Basingstoke, England) at 37°C for 24 h. *S. aureus *was identified using Vitek 2 with Gram-Positive (GP) cards (BioMérieux, Mary l'Etoile, France) following manufacturer's recommendations.

### Molecular typing and antibiotic resistance

The minimal inhibitory concentration (MIC) of the antibiotics was determined by broth microdilution in Müller-Hinton using the Sensititre susceptibility plate EUST (Trek Diagnostics Systems, East Grinstead, England; MCS Diagnostics BV, Swalmen, The Netherlands), except for spectinomycin, which was tested using homemade microbroth dilution plates. The MIC was interpreted according to the European Committee on Antimicrobial Susceptibility Testing (EUCAST) guidelines http://www.eucast.org using clinical resistance breakpoints for cefoxitin (> 4 mg/L), chloramphenicol (> 8 mg/L), clindamycin (> 0.5 mg/L), ciprofloxacin (> 1 mg/L), erythromycin (> 2 mg/L), fusidic acid (> 1 mg/L), gentamicin (> 1 mg/L), linezolid (> 4 mg/L), penicillin (> 0.125 mg/L), quinupristin/dalfopristin (> 2 mg/L), rifampicin (> 0.5 mg/L), tetracycline (> 2 mg/L), trimethoprim (> 4 mg/L), and vancomycin (> 2 mg/L). Otherwise, resistance breakpoints were tentatively derived from epidemiological MIC cut off values from EUCAST for kanamycin (> 8 mg/L), mupirocin (> 1 mg/L), streptomycin (> 16 mg/L), spectinomycin (> 128 mg/L), sulfamethoxazole (> 128 mg/L), and tiamulin (> 2 mg/L). Antibiotic resistance genes, including *mecA*, were detected using a microarray [45]. SCC*mec *types were determined by multiplex Polymerase Chain Reaction PCR assays [46]. Sequence types (ST) were determined using multilocus sequence typing (MLST) [31]. *Spa *type was determined as previously described and analysed using the Ridom StaphType software (Ridom StaphType, Ridom GmbH, Würzburg, Germany) [47]. The presence of the Panton-Valentine Leukocidin operon *lukS-lukF *was determined by PCR as described previously [48].

### Statistical Analysis

Sampling calculations were performed using the Win Episcope 2.0 software http://www.clive.ed.ac.uk/winepiscope. The difference in the prevalence between the years was analysed by Chi-square test. The level of significance was 5%. All statistics were performed using the NCSS 2007 statistical software (Hintze J. NCSS (2008) Version 07.1.8. Kaysville, UT, USA).

## Authors' contributions

GO wrote the manuscript, supervised the analyses for the detection and identification of MRSA and contributed to the conception of the study. SB designed the sampling plan, organised the sampling at the slaughterhouses and performed statistical analyses. AR carried out the molecular genetic studies. VP designed and coordinated the study, and helped to write the manuscript. All authors read and approved the final manuscript.

## References

[B1] Cuny C Friedrich A Kozytska S Layer F Nubel U Ohlsen K Emergence of methicillin-resistant *Staphylococcus aureus *(MRSA) in different animal species Int J Med Microbiol 2010 300 109 117 10.1016/j.ijmm.2009.11.002 20005777

[B2] Weese JS Methicillin-resistant *Staphylococcus aureus *in animals ILAR J 2010 51 233 244 21131724 10.1093/ilar.51.3.233

[B3] Vanderhaeghen W Hermans K Haesebrouck F Butaye P Methicillin-resistant *Staphylococcus aureus *(MRSA) in food production animals Epidemiol Infect 2010 138 606 625 10.1017/S0950268809991567 20122300

[B4] Smith TC Pearson N The emergence of *Staphylococcus aureus *ST398 Vector Borne Zoonotic Dis 2011 11 327 339 10.1089/vbz.2010.0072 20925523

[B5] Wagenaar JA Yue H Pritchard J Broekhuizen-Stins M Huijsdens X Mevius DJ Unexpected sequence types in livestock associated methicillin-resistant *Staphylococcus aureus *(MRSA): MRSA ST9 and a single locus variant of ST9 in pig farming in China Vet Microbiol 2009 139 405 409 10.1016/j.vetmic.2009.06.014 19608357

[B6] Guardabassi L O'Donoghue M Moodley A Ho J Boost M Novel lineage of methicillin-resistant *Staphylococcus aureus*, Hong Kong Emerg Infect Dis 2009 15 1998 2000 19961685 10.3201/eid1512.090378 PMC3044525

[B7] Vanderhaeghen W Cerpentier T Adriaensen C Vicca J Hermans K Butaye P Methicillin-resistant *Staphylococcus aureus *(MRSA) ST398 associated with clinical and subclinical mastitis in Belgian cows Vet Microbiol 2010 144 166 171 10.1016/j.vetmic.2009.12.044 20092969

[B8] Catry B van Duijkeren E Pomba MC Greko C Moreno MA Pyorala S Reflection paper on MRSA in food-producing and companion animals: epidemiology and control options for human and animal health Epidemiol Infect 2010 138 626 644 10.1017/S0950268810000014 20141646

[B9] Wulf M Voss A MRSA in livestock animals-an epidemic waiting to happen? Clin Microbiol Infect 2008 14 519 521 10.1111/j.1469-0691.2008.01970.x 18325034

[B10] van Loo I Huijsdens X Tiemersma E de Neeling A van de Sande-Bruinsma N Beaujean D Emergence of methicillin-resistant *Staphylococcus aureus *of animal origin in humans Emerg Infect Dis 2007 13 1834 1839 18258032 10.3201/eid1312.070384 PMC2876750

[B11] Mulders MN Haenen AP Geenen PL Vesseur PC Poldervaart ES Bosch T Prevalence of livestock-associated MRSA in broiler flocks and risk factors for slaughterhouse personnel in The Netherlands Epidemiol Infect 2010 138 743 755 10.1017/S0950268810000075 20109255

[B12] Khanna T Friendship R Dewey C Weese JS Methicillin resistant *Staphylococcus aureus *colonization in pigs and pig farmers Vet Microbiol 2008 128 298 303 10.1016/j.vetmic.2007.10.006 18023542

[B13] van Belkum A Melles DC Peeters JK van Leeuwen WB van Duijkeren E Huijsdens XW Methicillin-resistant and-susceptible *Staphylococcus aureus *sequence type 398 in pigs and humans Emerg Infect Dis 2008 14 479 483 10.3201/eid1403.0760 18325267 PMC2570802

[B14] Smith TC Male MJ Harper AL Kroeger JS Tinkler GP Moritz ED Methicillin-resistant *Staphylococcus aureus *(MRSA) strain ST398 is present in midwestern U.S. swine and swine workers PLoS ONE 2009 4 e4258 10.1371/journal.pone.0004258 19145257 PMC2626282

[B15] van Rijen MML van Keulen PH Kluytmans JA Increase in a Dutch Hospital of methicillin-resistant *Staphylococcus aureus *related to animal farming Clin Infect Dis 2008 46 261 263 10.1086/524672 18171259

[B16] van Rijen MM Bosch T Heck ME Kluytmans JA Meticillin-resistant *Staphylococcus aureus *epidemiology and transmission in a Dutch hospital J Hosp Infect 2009 72 299 306 10.1016/j.jhin.2009.05.006 19596488

[B17] Witte W Strommenger B Stanek C Cuny C Methicillin-resistant *Staphylococcus aureus *ST398 in humans and animals, Central Europe Emerg Infect Dis 2007 13 255 258 10.3201/eid1302.060924 17479888 PMC2725865

[B18] Potel C Alvarez-Fernandez M Constenla L Alvarez P Perez S First human isolates of methicillin-resistant *Staphylococcus aureus *sequence type 398 in Spain Eur J Clin Microbiol Infect Dis 2010 29 351 352 10.1007/s10096-009-0860-z 20094897

[B19] European Food Safety Authority Analysis of the baseline survey on the prevalence of methicillin resistant *Staphylococcus aureus *(MRSA) in holding with breeding pigs, in the EU, 2008, Part A: MRSA prevalence estimates EFSA J 2009 7 1376

[B20] Riesen A Perreten V Antibiotic resistance and genetic diversity in *Staphylococcus aureus *from slaughter pigs in Switzerland Schweiz Arch Tierheilk 2009 151 425 431 10.1024/0036-7281.151.9.425 19722130

[B21] Nitzsche S Zweifel C Stephan R Phenotypic and genotypic traits of *Staphylococcus aureus *strains isolated from pig carcasses Vet Microbiol 2007 120 292 299 10.1016/j.vetmic.2006.10.027 17141430

[B22] Huber H Koller S Giezendanner N Stephan R Zweifel C Prevalence and characteristics of meticillin-resistant *Staphylococcus aureus *in humans in contact with farm animals, in livestock, and in food of animal origin, Switzerland, 2009 Euro Surveill 2010 15 1 4 20430001

[B23] Büttner S Kuhn M Antibiotikaresistenzmonitoring-Jahresbericht 2008 http://www.bvet.admin.ch/themen

[B24] Jung YH Shin ES Kim O Yoo JS Lee KM Yoo JI Characterization of two newly identified genes, *vgaD *and *vatG*, conferring resistance to streptogramin A in *Enterococcus faecium* Antimicrob Agents Chemother 2010 54 4744 4749 10.1128/AAC.00798-09 20713681 PMC2976166

[B25] Long KS Poehlsgaard J Kehrenberg C Schwarz S Vester B The Cfr rRNA methyltransferase confers resistance to Phenicols, Lincosamides, Oxazolidinones, Pleuromutilins, and Streptogramin A antibiotics Antimicrob Agents Chemother 2006 50 2500 2505 10.1128/AAC.00131-06 16801432 PMC1489768

[B26] Miller K Dunsmore CJ Fishwick CW Chopra I Linezolid and tiamulin cross-resistance in *Staphylococcus aureus *mediated by point mutations in the peptidyl transferase center Antimicrob Agents Chemother 2008 52 1737 1742 10.1128/AAC.01015-07 18180348 PMC2346621

[B27] Hooper DC Mechanisms of action and resistance of older and newer fluoroquinolones Clin Infect Dis 2000 31 Suppl 2 S24 S28 10984324 10.1086/314056

[B28] Grundmann H Aanensen DM van den Wijngaard CC Spratt BG Harmsen D Friedrich AW Geographic distribution of *Staphylococcus aureus *causing invasive infections in Europe: a molecular-epidemiological analysis PLoS Med 2010 7 e1000215 10.1371/journal.pmed.1000215 20084094 PMC2796391

[B29] Sakwinska O Kuhn G Balmelli C Francioli P Giddey M Perreten V Genetic diversity and ecological success of *Staphylococcus aureus *strains colonizing humans Appl Environ Microbiol 2009 75 175 183 10.1128/AEM.01860-08 18978084 PMC2612194

[B30] Megevand C Gervaix A Heininger U Berger C Aebi C Vaudaux B Molecular epidemiology of the nasal colonization by methicillin-susceptible *Staphylococcus aureus *in Swiss children Clin Microbiol Infect 2010 16 1414 1420 10.1111/j.1469-0691.2009.03090.x 19845693

[B31] Enright MC Day NP Davies CE Peacock SJ Spratt BG Multilocus sequence typing for characterization of methicillin-resistant and methicillin-susceptible clones of *Staphylococcus aureus* J Clin Microbiol 2000 38 1008 1015 10698988 10.1128/jcm.38.3.1008-1015.2000 PMC86325

[B32] Simpson V Davison N Hudson L Whatmore AM *Staphylococcus aureus *ST49 infection in red squirrels Vet Rec 2010 167 69 20622214 10.1136/vr.c3625

[B33] Otter JA French GL Molecular epidemiology of community-associated meticillin-resistant *Staphylococcus aureus *in Europe Lancet Infect Dis 2010 10 227 239 10.1016/S1473-3099(10)70053-0 20334846

[B34] Lewis HC Mølbak K Reese C Aarestrup FM Selchau M Sørum M Pigs as source of methicillin-resistant *Staphylococcus aureus *CC398 infections in humans, Denmark Emerg Infect Dis 2008 14 1383 1389 10.3201/eid1409.071576 18760004 PMC2603104

[B35] Aspiroz C Lozano C Vindel A Lasarte JJ Zarazaga M Torres C Skin lesion caused by ST398 and ST1 MRSA, Spain Emerg Infect Dis 2010 16 157 159 20031071 10.3201/eid1601.090694 PMC2874358

[B36] Cuny C Strommenger B Witte W Stanek C Clusters of infections in horses with MRSA ST1, ST254, and ST398 in a veterinary hospital Microb Drug Resist 2008 14 307 310 10.1089/mdr.2008.0845 19025385

[B37] Battisti A Franco A Merialdi G Hasman H Iurescia M Lorenzetti R Heterogeneity among methicillin-resistant *Staphylococcus aureus *from Italian pig finishing holdings Vet Microbiol 2010 142 361 366 10.1016/j.vetmic.2009.10.008 19914010

[B38] Juhasz-Kaszanyitzky E Janosi S Somogyi P Dan A van der Graaf-van Bloois van Duijkeren E MRSA transmission between cows and humans Emerg Infect Dis 2007 13 630 632 10.3201/eid1304.060833 17553285 PMC2725960

[B39] Broens EM Graat EAM van der Wolf PJ van de Giessen AW De Jong MCM Transmission of methicillin resistant *Staphylococcus aureus *among pigs during transportation from farm to abattoir Vet J 2010 10.1016/j.tvjl.2010.08.003 20850359

[B40] de Neeling AJ van den Broek MJM Spalburg EC van Santen-Verheuvel MG Dam-Deisz WDC Boshuizen HC High prevalence of methicillin resistant *Staphylococcus aureus *in pigs Vet Microbiol 2007 122 366 372 10.1016/j.vetmic.2007.01.027 17367960

[B41] Hasman H Moodley A Guardabassi L Stegger M Skov RL Aarestrup FM Spa type distribution in *Staphylococcus aureus *originating from pigs, cattle and poultry Vet Microbiol 2010 141 326 331 10.1016/j.vetmic.2009.09.025 19833458

[B42] Tenhagen BA Fetsch A Stuhrenberg B Schleuter G Guerra B Hammerl JA Prevalence of MRSA types in slaughter pigs in different German abattoirs Vet Rec 2009 165 589 593 10.1136/vr.165.20.589 19915190

[B43] Harbarth S Hawkey PM Tenover F Stefani S Pantosti A Struelens MJ Update on screening and clinical diagnosis of meticillin-resistant *Staphylococcus aureus *(MRSA) Int J Antimicrob Agents 2011 37 110 117 10.1016/j.ijantimicag.2010.10.022 21163628

[B44] Lozano C Aspiroz C Lasarte JJ Gomez-Sanz E Zarazaga M Torres C Dynamic of nasal colonization by methicillin-resistant *Staphylococcus aureus *ST398 and ST1 after mupirocin treatment in a family in close contact with pigs Comp Immunol Microbiol Infect Dis 2011 34 e1 e7 10.1016/j.cimid.2010.06.006 20663559

[B45] Perreten V Vorlet-Fawer L Slickers P Ehricht R Kuhnert P Frey J Microarray-based detection of 90 antibiotic resistance genes of gram-positive bacteria J Clin Microbiol 2005 43 2291 2302 10.1128/JCM.43.5.2291-2302.2005 15872258 PMC1153730

[B46] Kondo Y Ito T Ma XX Watanabe S Kreiswirth BN Etienne J Combination of multiplex PCRs for Staphylococcal Cassette Chromosome *mec *type assignment: rapid identification system for *mec, ccr*, and major differences in junkyard regions Antimicrob Agents Chemother 2007 51 264 274 10.1128/AAC.00165-06 17043114 PMC1797693

[B47] Harmsen D Claus H Witte W Rothgänger J Claus H Turnwald D Typing of methicillin-resistant *Staphylococcus aureus *in a university hospital setting by using novel software for *spa *repeat determination and database management J Clin Microbiol 2003 41 5442 5448 10.1128/JCM.41.12.5442-5448.2003 14662923 PMC309029

[B48] Lina G Piémont Y Godail-Gamot F Bes M Peter M-O Gauduchon V Involvement of Panton-Valentine leukocidin-producing *Staphylococcus aureus *in primary skin infections and pneumonia Clin Infect Dis 1999 29 1128 1132 10.1086/313461 10524952

